# Associations of tobacco smoking with body mass distribution; a population-based study of 65,875 men and women in midlife

**DOI:** 10.1186/s12889-019-7807-9

**Published:** 2019-11-01

**Authors:** Sidsel Graff-Iversen, Stephen Hewitt, Lisa Forsén, Liv Grøtvedt, Inger Ariansen

**Affiliations:** 1 Division of Mental and Physical Health, Norwegian Institute of Public Health, Oslo, Norway; 2 Department of Endocrinology, Morbid Obesity and Preventive Medicine, Medical Clinic, Oslo University Hospital Aker, Oslo, Norway; 3 National Resource Centre for Women’s Health, Division of Obstetrics and Gynaecology, Rikshospitalet, Oslo University Hospital, Oslo, Norway

**Keywords:** Smoking, Abdominal obesity, Waist circumference, Hip circumference, Waist-hip ratio

## Abstract

**Background:**

Studies indicate an effect of smoking toward abdominal obesity, but few assess hip and waist circumferences (HC and WC) independently. The present study aimed to assess the associations of smoking status and volume smoked with HC and WC and their ratio in a population with low prevalence of obesity together with high prevalence of smoking.

**Methods:**

We used cross-sectional survey data from 11 of a total 19 Norwegian counties examined in 1997–99 including 65,875 men and women aged 39–44 years. Analysis of associations were adjusted for confounding by socioeconomic position, health indicators, and additionally for BMI.

**Results:**

Compared with never-smokers, when adjusting for confounders and in addition for BMI, mean HC remained lower while mean WC and waist-hip-ratio (WHR) were higher in current smokers. The finding of a lower HC and higher WHR level among smokers was consistent by sex and in strata by levels of education and physical activity, while the finding of higher WC by smoking was less consistent. Among current smokers, BMI-adjusted mean HC decreased whereas WC and WHR increased by volume smoked. Compared with current smokers, former smokers had higher BMI-adjusted HC, lower WHR and among women WC was lower.

**Conclusions:**

The main finding in this study was the consistent negative associations of smoking with HC. In line with the hypothesis that lower percentage gluteofemoral fat is linked with higher cardiovascular risk, our results suggest that smoking impacts cardiovascular risk through mechanisms that reduce the capacity of fat storage in the lower body region.

## Background

Fat accumulation around visceral organs seems associated with higher risk of cardiovascular diseases (CVD) and diabetes than a more globally distribution of body fat [1, 2]. Abdominal obesity and smoking are both linked with an unhealthy metabolic risk profile [3–5], giving a synergy of risk on CVD. Nearly all studies show that smoking is associated with lower body mass index (BMI) [6, 7], which by itself might be considered a healthy effect. But several studies show positive associations of current smoking with waist-hip-ratio (WHR) [5, 8], concluding that smoking enhances abdominal obesity as an unhealthy outcome. Also, a study that used a Mendelian randomisation approach indicated a causal effect of tobacco smoking on abdominal fat accumulation [9].

Of earlier studies, some [5, 8], but not all studies have analysed the association of smoking with WHR and waist circumference (WC) with adjustment for BMI. In one study, current smoking was negatively associated with WC, whereas additional adjustment for BMI changed the direction into a positive association [10]. Another study concluded that mean WC was lower in smokers, but that the level increased with the volume smoked [11]. Knowledge and theories on biological pathways from smoking to changes in body fat volume, deposition and type of fat**,** include hormonal and metabolic mechanisms [7] and the influence of smoking on the intestinal microbiome [12, 13].

Most people gain weight after smoking cessation [14, 15]. In a large population-based follow-up study, weight-gain after smoking cessation was associated with increased short-term risk of diabetes, but the gain did not mitigate benefits on CVD and all-cause mortality [16]. Several biological processes might explain the weight gain [5, 7], but the mechanism by which tobacco cessation leads to impaired glucose metabolism is not clear [15]. In spite of the long-term health benefits of smoking cessation, weight concern seems to be a reason to continue smoking and to relapse after cessation [14]. A recent Mendelian randomization study found that the volume smoked was positively associated with overweight [17], suggesting that overweight causally influence smoking behaviour. In the perspective of the overweight epidemic [18], together with an estimated 1.1 billion current tobacco smokers by 2025 [19], a comorbidity setting of overweight and tobacco use may be increasingly relevant as a public health and clinical challenge. In this study, the aim was to investigate how smoking characteristics were associated with indicators of body mass distribution in a large population in their early 40ies, known to have high smoking prevalence, early smoking debut, and relatively low prevalence of obesity [20, 21].

## Methods

### Study population

Health surveys were performed in Norwegian counties during 1985–99 as part of the National Health Screening Service’s Age 40 Program, inviting all inhabitants who completed 40–42 years during the year the survey started in their county of living [20, 21]. This design implies an age range of 39–44 years by the time of examination. The age around 40 was chosen for the purpose of CVD prevention, as the participants were informed on their cardiovascular risk and advised on modification of risk factors [21]. In the period 1997–99, surveys were conducted in 11 of a total of 19 Norwegian counties. The age range 40–42 years was extended in one county to 40–43 years and in one small municipality to 37–44 years, hence 132 persons fell outside the age range of 39–44 years by examination and were excluded.

The invitation was sent by a postal letter, including the main survey questionnaire. A second postal invitation was sent to primary non-participants. Prior to the health examination, the participants filled in the main questionnaire which covered subjective health in general, history of diabetes, myocardial infarction, angina pectoris and stroke, tobacco smoking, leisure-time physical activity (LTPA), alcohol use, educational level, disability pension and a few other items.

In all 103,668 men and women were invited. Of these, 66,744 (64%) met for examination and allowed the use of data for research. Exclusions were made due to missing values of anthropometric measures (*n* = 261), age outside the range 39–44 years when examined (*n* = 132), or inconsistent information on smoking status (*n* = 493) by giving negative report on current or past daily smoking, but reporting on volume smoked or years as a smoker. As occasional smoking was not asked for, we could not differentiate occasional smokers from those with non-logical reports, and this group was excluded. The remaining 65,875 (30,579 men and 35,296 women) were included in analysis.

### Smoking status

Questions on current daily smoking (cigarettes, cigars, cigarillos and pipe separately) were to be answered by yes or no, followed by a statement “Never smoked daily” to be confirmed or not. Smokers reported the age of smoking debut and total number of years as a smoker. There was no categorical question on former smoking, but one question was worded “If you have smoked daily earlier, how many years ago did you quit?” Occasional smoking was not recorded. There was no biochemical validation of the self-report on smoking, but a comparable questionnaire in Finnish surveys was validated [22]. For analysis, participants who confirmed the statement “never smoked daily” as their only report on tobacco use were included as “never-smokers”. “Current smokers” were defined by any report of current daily smoking. “Former smokers” were defined by report of number of years as a daily smoker, together with negative report on current daily smoking. We used the reported number of cigarettes per day as a continuous variable and constructed three ordinal groups of 1–9, 10–19, and ≥ 20 cigarettes per day to indicate the volume smoked: “light”, “intermediate” and “heavy”. These ordinal groups were of reasonable size in both genders, as expected from an earlier Norwegian study [23].

### Anthropometric measurements

Height and weight were measured with light clothes and without shoes by use of an electronic device (DS-102, Arctic Heating AS, Norway, produced by Dong Sahn Jenix & Co, Ltd., Seoul, Korea), rounded to the nearest cm and 0.1 kg. It was validated by a standard load of 60 kg. Relative body weight was measured by BMI, calculated as weight in kg divided by height in meter squared [1]. Hip circumference (HC) and WC were measured in cm in standing position with a flexible, not stretchable, steel device. HC was recorded at the hip at its widest and WC at the level of umbilicus or, in obese persons, at the widest between the lowest rib and the iliac crest. WHR was calculated by dividing WC with HC. For determining the cuff size for blood pressure measurement, the mid-upper arm circumference (MUAC) was registered at its maximum in cm with one decimal. MUAC has been proposed as an easily applicable indicator of overweight [24].

### Indicators of socioeconomic position

Educational attainment was considered as a confounding factor, associated with both smoking and anthropometric measures [25, 26], and was assessed by self-report according to five levels. For stratified analysis we defined three educational levels: basic (until 10 years), secondary (1–4 years after basic), and tertiary (more than 4 years completed education after basic). Disability pension was self-reported and used, together with measured body height [27], as additional indicators of socioeconomic position.

### Other covariates

We assessed LTPA by two different questions [28]. One question asked for recreational activities during a usual week, including commuting to work, with one of four answering alternatives: (1) “Read, watch TV or other sedentary activities”; (2) “Walk, cycle or other bodily movement, including commuting to work, at least 4 hours per week”; (3) “Recreational sport or heavy garden work at least 4 hours per week”; (4) “Regular sport training or competitions several times a week”. The other question made it possible to quantify vigorous LTPA from null up to 3 h or more per week. For analysis we combined these questions into three ordinal groups: “sedentary” (lowest alternative on the first questionnaire and < 1 h vigorous LTPA), “moderately active” (alternative 2 on the first questionnaire or 1–2 h vigorous LTPA) and “highly active” (alternatives 3 or 4 on the first questionnaire or > 3 h vigorous LTPA). Both these short questionnaires, the first in particular, have been indirectly validated [28, 29].

Alcohol use was assessed by questions on total abstaining, frequency of alcohol intake during a usual month, and number of drinks during two weeks. For analysis we grouped alcohol intake into “never”, “less than 10 drinking occasions per month”, and “>10 occasions per month”. Dietary habits were assessed by a very brief questionnaire with four answering alternatives, including (1) “fish”, (2) “fruit/ vegetables”, (3)” full-fat milk”, “low-fat milk”, “skimmed milk”, and (4) “type of fat used on bread and for cooking” [20].

### Analysis

We assessed the association of current smoking with BMI, HC, WC, WHR and MUAC by comparing with never-smokers, and by comparing current with former smokers, aware of a potential over-representation of people with overweight issues among the current smokers, as overweight might be a barrier to smoking cessation. To assess the hypothesis that smoking is linked with abdominal obesity within levels of BMI, we adjusted for BMI to disentangle the more direct effect of smoking on HC and WC. The inconsistency in earlier studies, some making adjustment for BMI and others not, was a reason for presenting models with adjustment for typical confounders and for BMI additionally. This choice was in accordance with theories that nicotine, via its impact on cortisol, epinephrine and sex hormone levels [7], has diverse effects on abdominal and gluteal fat.

We used multiple analysis of variance for comparing mean values. The models included Model 1_Observed,_ Model 2_SEP and health indicators_ adjusted for body height, education, disability pension, LTPA and alcohol use, and Model 3_BMI additionally,_ with additional adjustment for BMI. We did not adjust for self-reported health, nor self-reported CVD, as health problems might be results of smoking. We also assessed the associations between each anthropometric measure and smoking status by Pearson correlation coefficients and linear regression. Interactions between smoking status and education and between smoking status and LTPA levels were assessed by univariate analysis of variance in Model 2_SEP and health indicators_ for the associations of current versus never smoking with anthropometric measures. All data analyses were performed with the statistical package SPSS version 16.0 (SPSS Inc., Chicago IL).

## Results

Mean age was 41.2 ± SD 1.1 years in men and women; 10.5% were 43 to 44 years, and 1.5% were 39 years. Of the men, 35% were current smokers, 40% were never-smokers, and 25% were former smokers, and in women these figures were 38, 37 and 25%, respectively. Among current smokers, 99% of the men and 100% of the women smoked cigarettes. The mean age of smoking debut was 17 years in both sexes, with practically all debuts within 11 to 22 years. Mean years of daily smoking was 22.0 ± 5.1 years in current smokers and 12.3 ± 7.0 years in former smokers, with minimal difference by sex. In all, 642 men (2.1%) and 495 women (1.4%) reported diabetes, and/or a history of myocardial infarction, angina or stroke.

Additional file 1 Table S1 shows unadjusted values of SEP, health behaviors and other CVD risk factors, together with anthropometric measures, by smoking status. In both sexes, mean weight, BMI, HC, WC and MUAC values were lowest among current smokers and highest among former smokers, while WHR showed minimal differences by smoking status. The correlation of BMI with HC, WC and MUAC was high, with correlation coefficients > 0.8 in all subgroups by smoking status, while the correlation of BMI with WHR was 0.5 (results not shown).

### Anthropometric measures in current versus never-smokers

Table 1 shows mean values of anthropometric measures comparing current smokers with never-smokers. The inclusion of confounders in Model 2_SEP and health indicators_ accentuated a trend of lower BMI, HC, WC and MUAC in smokers. By additional adjustment for BMI, the differences in HC diminished, and the pattern of WC changed from lower to higher level in current smokers than never-smokers. In this model mean WHR was minimally higher in smokers, while MUAC showed no or minimal difference (Model 3_BMI additionally_), see Table 1. Adjustment for age, county and dietary factors (fish and vegetable/ fruit consumption) did not change the observed results and were not included in the final models.

**Table 1:** Mean values of anthropometric measurements by current versus never smoking

Smoking status	Model 1_Observed_		Model 2_SEP and health indicators_		Model 3 _BMI additionally_	
	Never	Current	Never	Current	Never	Current
*Men, n*	*12,199*	*10,770*				
BMI, kg/m^2^	26.6	26.1***	26.7	26.0***		
HC, cm	103.2	101.7***	103.2	101.8***	102.7	102.2***
WC, cm	91.8	90.8***	92.0	90.6***	91.2	91.4**
WHR	0.89	0.89***	0.89	0.89 ^NS^	0.888	0.893***
MUAC, cm	31.0	30.8***	31.1	30.7***	30.8	30.9 ^NS^
*Women, n*	*13,002*	*13,350*				
BMI, kg/m^2^	25.2	24.7***	25.3	24.6***		
HC, cm	101.4	99.9***	101.6	99.7***	100.9	100.4***
WC, cm	78.8	78.6^p = 0.07^	79.2	78.2***	78.4	79.0***
WHR	0.78	0.79***	0.78	0.78***	0.78	0.79***
MUAC, cm	28.6	28.2***	28.6	28.1***	28.4	28.3***

*N* = 30,579 men, 35,296 women. Model 1_Observed_; Model 2_SEP and health indicators_, adjusted for body height, education, disability pension, physical activity and alcohol use. Model 3_BMI additionally,_ adjusted for education, disability pension, physical activity, alcohol use and BMI. **p < 0.01; *** *p* < 0.001

### Anthropometric measures by volume smoked

**Fig. 1:**
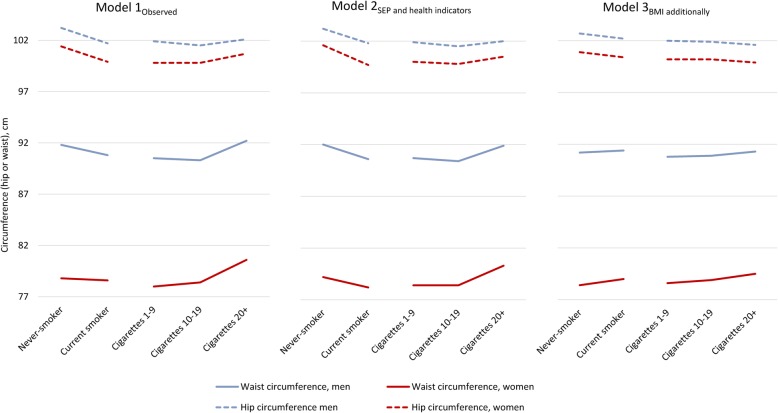
Observed and adjusted mean hip and waist circumferences by smoking status (never, current) and current volume smoked (1–9 cigarettes, 10–19 cigarettes, 20 cigarettes or more). Based on Tables 1 and 2

Table 2 shows mean values of the anthropometric measures in current cigarette smokers grouped by volume smoked. When adjusting for confounders (Model 2_SEP and health indicators_), we found a pattern with lowest BMI and HC (both sexes) by intermediate cigarette consumption, and for WC in men. All anthropometric measures, including WHR, were in both sexes highest by the highest volume smoked (Model 2_SEP and health indicators_). With additional adjustments for BMI, HC decreased, while WC and WHR increased by volume smoked (Model 3_BMI additionally_). Fig. 1 shows mean values of HC and WC in current cigarette smokers and never-smokers, to the left grouped by smoking status corresponding to Table 1, and to the right by number of cigarettes smoked per day, corresponding to Table 2. Linear regression analyses did not substantially change these results.

**Table 2:** Mean values of anthropometric measures in current cigarette smokers by volume smoked^1^

Cigarettes/day	Model 1 _Observed_			Model 2_SEP and health indicators_			Model 3 _BMI additionally_		
	1–9	10–19	20+	1–9	10–19	20+	1–9	10–19	20+
*Men, n*	*2105*	*5965*	*2530*						
BMI, kg/m^2^	26.0	25.9	26.4***	26.1	25.9	26.4***			
HC, cm	101.9	101.5	102.1***	101.9	101.5	102.0**	102.0	101.9	101.6***
WC, cm	90.5	90.3	92.2***	90.7	90.4	91.9***	90.8	90.9	91.3**
WHR	0.89	0.89	0.90***	0.89	0.89	0.90***	0.89	0.89	0.90***
*Women, n*	*4082*	*7648*	*1545*						
BMI, kg/m^2^	24.6	24.7	25.3***	24.7	24.6	25.2***			
HC, cm	99.8	99.8	100.7***	100.0	99.8	100.5**	100.2	100.2	99.9*
WC, cm	78.0	78.4	80.6***	78.4	78.4	80.3***	78.6	78.9	79.5***
WHR	0.78	0.78	0.80***	0.78	0.78	0.80***	0.78	0.79	0.79***

*N* = 23,875; 10,600 men, 13,275 women. ^1^Volume smoked is defined by number of cigarettes per day

Model 1_Observed_, adjusted for age and county; Model 2_SEP and health indicators_, adjusted for body height, education, disability pension, physical activity and alcohol use. Model 3_BMI additionally,_ adjusted for education, disability pension, physical activity, alcohol use and BMI. **p < 0.01; *** *p* < 0.001

### Stratified analyses

**Fig. 2:**
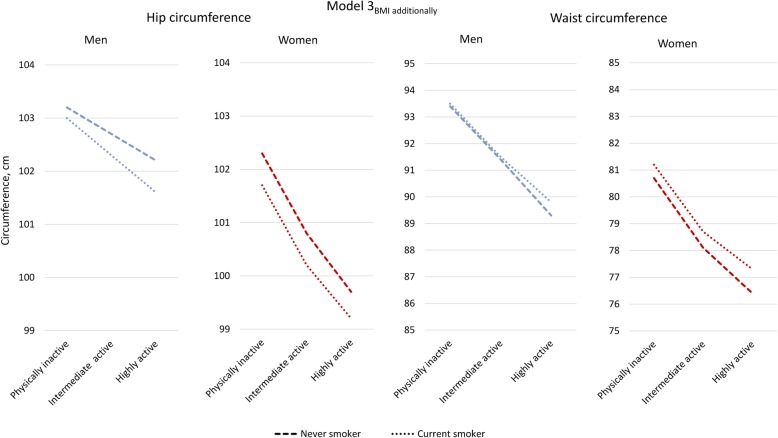
Adjusted mean hip and waist circumferences by leisure time physical activity level in never and current smokers. Based on Additional file 2: Table S2

The interaction term for smoking with LTPA was significant (*p* < 0.05 or higher) for all the anthropometric measures, and we performed stratified analyses (Additional file 2: Table S2). In all strata by LTPA, the adjusted values of BMI, HC and WC were lower in current smokers compared to never-smokers, except for no significant difference in mean WC among the most active women. With additional adjustment for BMI, HC remained consistently lower in smokers, while mean WC values changed to consistently higher in smokers among women in all strata and among the most active men. Figure 2 shows BMI-adjusted mean levels of HC and WC in the ordinal groups by LTPA, in never-smokers and current smokers. The levels of confounder adjusted WC were markedly lower among physically highly active compared to inactive; among the current smokers by approximately 3 cm and among never-smokers by around 4 cm. Adjusted mean WHR showed small if any difference by smoking (Model 2_SEP and health indicators_), however with slightly higher values by current smoking when adjusting for BMI (Additional file 2: Table S2).

Interaction terms of smoking with education was significant (*p* <0.001) for BMI, HC, WC and WHR. We performed stratified analysis by educational level (Additional file 3: Table S3). Among basic and secondary educated, current smokers had lower BMI than had never-smokers, while there were minimal differences among the tertiary educated men and women (Model 2 _SEP and health indicators_). In all strata, current smokers had lower adjusted HC than never-smokers. Among the basic and secondary educated men and women, also mean WC values were lower in smokers (Model 2 _SEP and health indicators_). When adjusting for BMI, mean WC did not differ by smoking among basic and secondary educated men, while among tertiary educated men, and among women in all strata, the pattern changed into higher mean WC in current smokers. In tertiary educated women the pattern of higher mean WC in current smokers was present both in the confounder-adjusted and the BMI-adjusted analyses.

In both sexes WHR was slightly higher by smoking in all strata (Model 3_BMI additionally_). In contrast, MUAC showed minimal differences by smoking when adjusting for BMI (not shown). Additional file 4: Figure S1 shows the BMI-adjusted mean values of HC and WC in current smokers and never-smokers in strata by educational attainment.

### Anthropometric measures in former versus current smokers

Table 3 shows that adjusted mean values of BMI, HC and WC were lower in current smokers compared with former smokers, while WHR was lower by current smoking in men only and did not differ by smoking in women (Model 2_SEP and health indicators_). With additional adjustment for BMI, mean HC remained lower by current smoking. Mean WHR was higher by current than former smoking in both sexes, and in women also mean WC was higher (Model 3_BMI additionally_).

**Table 3:** Mean values of anthropometric measures by former versus current smoking

Smoking status	Model 1_Observed_		Model 2_SEP and health indicators_		Model 3 _BMI additionally_	
	Current	Former	Current	Former	Current	Former
*Men, n = 18,380*	*10,770*	*7610*				
BMI, kg/m^2^	26.1	27.1***	26.0	27.1***		
HC, cm	101.7	103.8***	101.8	103.7***	102.6	103.0***
WC, cm	90.8	93.2***	90.8	93.2***	92.0	92.0^NS^
WHR	0.89	0.90***	0.89	0.90***	0.90	0.89***
*Women, n = 22,294*	*13,350*	*8944*				
BMI, kg/m^2^	24.7	25.5	24.6	25.6		
HC, cm	99.9	101.8***	99.8	101.9***	100.7	101.0***
WC, cm	78.6	79.6***	78.3	79.6***	79.4	78.8***
WHR	0.79	0.78***	0.78	0.78^NS^	0.79	0.78***

*N* = 40,674. Model 1_Observed_; Model 2_SEP and health indicators_, adjusted for body height, education, disability pension, physical activity and alcohol use. Model 3_BMI additionally,_ adjusted for education, disability pension, physical activity, alcohol use and BMI. **p < 0.01; *** *p* <0.001

## Discussion

The results are in line with a hypothesis of tobacco smoking leading to loss of body mass around the hip, more than proportional to a lower BMI, together with a less than proportional loss around the waist. When adjusting for BMI, current smokers exhibited a pattern with lower HC and higher WC with increasing volume of cigarettes smoked. And, compared with former smokers, current smokers had smaller HC, and in women larger WC. In stratified analyses, the associations of smoking with HC were more consistent than the associations with WC.

### Strengths and limitations

This study is based on health examination surveys among all mid-life men and women in 11 of 19 Norwegian counties, and with a participation rate of 64%. Data collection was performed during 1997–99, a time when smoking was still common in all social strata, and the overweight epidemic was in an early phase.

A time-relationship of smoking debut preceding the anthropometric measurements was documented by self-reports. However, a tendency of selective smoking cessation must be counted upon, with weight concerns being a barrier to smoking cessation [14]. Such selection has probably affected our results toward camouflaging some of the difference between current and former smokers on BMI, HC and WC.

The questionnaire did not include occasional smoking as the health consequences of “light-intermittent” smoking were not recognised by the time of the survey. In retrospect, this choice is regrettable. According to another Norwegian study, occasional smokers had BMI and WC values between daily- and never-smokers [30]. If some non-daily smokers are included among never or former smokers, the differences in anthropometric measures by smoking status are likely to be underestimated. In our study, this problem was reduced by the exclusion of nearly 500 persons who did not confirm daily smoking, but reported on other smoking-related questions. Biochemical validation of the reports on smoking was not included in the survey. However, in the FINRISK-92 study, associations of self-reported smoking with serum cotinine was high**,** across strata of age, gender and socio-economic groups [22]. Still, there was probably some reporting bias, such as under-reporting the number of cigarettes per day.

The two LTPA questionnaires we used have been indirectly validated by correlations with metabolic biomarkers, physical fitness and resting pulse rate, indicating a reasonable validity [28, 29]. The questionnaire on diet was limited to only a few questions with few alternatives on frequency. A meaningful stratified analysis by dietary patterns could not be done, and the control for diet may be insufficient. A meta-analysis concluded that smokers had higher intake of energy, total fat, and alcohol than non-smokers, together with lower intake of polyunsaturated fat, fibre, vitamins C and E and beta-carotene [31]. However, another study with extensive control for diet found no difference between smokers and never-smokers in energy intake, but smokers had a less healthy diet with respect to CVD risk [32]. Of dietary factors, there are indications that constituents in the Mediterranean diet are of particular interest to reduce central obesity [33].

### Patterns by physical activity, educational attainment and sex

The trend of lower HC in smokers than never-smokers was consistent in strata by LTPA and educational attainment, while differences in WC by smoking did not reach statistical significance in all strata. The analysis of anthropometric measures by smoking status in strata by LTPA, revealed that the BMI-adjusted HC and WC levels were considerably higher among the physically inactive compared with the more active. This could be due to “reverse causation” with overweight being a reason for physical inactivity, but could also support hypotheses on the immune system to be responsive to physical exercise, with consequences for systemic inflammation and diversity of the gut microbiota [34], opposite to the effect of smoking [12]. This finding is in line with a study concluding that sedentary behaviour, in particular television viewing, is positively associated with visceral abdominal fat, intermuscular adipose tissue and liver fat, while physical activity had the opposite association [35].

The finding of a larger BMI-adjusted WC in smokers than non-smokers was more consistent in women than in men. A sex specific effect of smoking could be mediated by a number of mechanisms linked to sex chromosomes, sex hormones and earlier menopause in women who smoke [7, 36]. In the Oslo Health Study 2000–2001, tobacco smoking was linked with an earlier menopause in a dose-dependent manner with number of cigarettes per day and total tobacco exposure [37].

The finding of lower mean BMI in current smokers compared with never-smokers among basic and secondary educated men and women was expected [5–11], but the minimal differences among tertiary educated was unexpected. Interestingly, the highly educated smokers had BMI levels close to those of never-smokers and close to secondary educated smokers, in contrast to the expected lower BMI by smoking [6, 7] and the expected social gradient [25]. One explanation could be that highly educated people were particularly prone to smoke due to weight issues [14, 15], in line with a hypothesis of overweight being a barrier to smoking cessation.

### Smoking, body mass and body mass distribution

According to a review, most of the effect of smoking on body weight and fat deposits is likely to be mediated through nicotine [7]. Another review found evidence for specific effects of cigarette smoking on adipose tissues, such as differentiation of adipocytes, lipolysis and secretion properties [38]. A smoking cessation study found that cessation was linked with increased muscle mass, muscle strength and bone density, but primarily with accumulating extra fat in the gluteal region [39]. Our findings might support the hypothesis that tobacco smoking has a specific effect, particularly by reducing the fat deposition in the hip region, while smoking cessation seems to reverse this effect. Several studies indicate that gluteal and femoral fat have protective effects against diabetes and cardiovascular disease, independent of abdominal fat [40]. A review by Cameron et al. concluded that WC and HC considered together, but not as a ratio, may improve risk prediction models [41]. The findings in these studies have been supported by a recent follow-up study from the Women’s Health Initiative, concluding that both elevated abdominal fat and reduced leg fat are associated with increased risk of CVD [42]. One explanation could be that trapping of excess fatty acids in the gluteal region protects the cardiovascular system from exposure to elevated lipids [42]. One may, however, question if a larger volume of gluteal fat is protective by itself, or by the capacity of fat storage on a safer place in case of over-nutrition.

The finding in this study of a dose-response relationship of volume smoked with WC and HC may be of interest to hypotheses on how smoking affects the development of CVD. Potentially, lowering rates of smoking [19] has leaded to larger hips with healthier storage of free fatty acids. This might in part explain the “paradox” of lower incidence of myocardial infarction [43] in spite of the rising figures of overweight [18]. One of the links between smoking and overweight could be through effects on the gut microbiome, as smoking seems to reduce its microbiological diversity [12], while physical exercise acts in the opposite direction [34]. Lower diversity of the gut flora has consequences for gut inflammation which may impact on host metabolism, and in the end lead to the development and perpetuation of overweight [44]. In this perspective, the findings in our study may be of interest in light of emerging research on biological pathways from smoking and physical activity to changes in body fat storage and metabolism [12, 13, 34, 44].

## Conclusion

The main finding in this study was the consistent negative associations of smoking with HC. In perspective of research concluding that a higher percentage gluteofemoral fat is linked with lower CVD risk, our results suggest that smoking could be a modifying CVD risk factor through mechanisms that reduce the capacity of fat storage in the lower body region.

## Supplementary information

**Additional file 1: Table S1.** Socioeconomic factors, lifestyle indicators and anthropometric measurements by smoking status. *N* = 65,875.

**Additional file 2: Table S2.** Mean waist circumference and mean waist-hip ratio by smoking status by levels of leisure-time physical activity.

**Additional file 3: Table S3.** Mean values of weight- related indices by smoking status by educational attainment.

**Additional file 4: Figure S1.** Adjusted mean hip and waist circumferences by smoking status in strata by education. Based on Additional file 3: Table S3.

## Data Availability

The dataset generated during the current study is not publicly available, but may be available to research groups on request to the Norwegian Institute of Public health.

## Abbreviations

BMI: body mass index
CVD: cardiovascular disease
HC: hip circumference
LTPA: leisure-time physical activity
MUAC: mid-upper arm circumference
SPSS: Statistical Package for Social Sciences
WC: waist circumference
WHR: waist-hip ratio
