# Supplementary material for: Associations of tobacco smoking with body mass distribution; a population-based study of 65,875 men and women in midlife
Source: BMC Public Health. 2019 Nov 1;19:1439. doi: 10.1186/s12889-019-7807-9 (PMC6825363; doi:10.1186/s12889-019-7807-9)
Supplement: Supplementary file 1 — Additional file 1: Table S1. Socioeconomic factors, lifestyle indicators and anthropometric measurements by smoking status. N = 65,875. [file 12889_2019_7807_MOESM1_ESM.docx]

Additional file 1: Table S1. Socioeconomic factors, lifestyle indicators and anthropometric measurements by smoking status. N=65,875

|  | Men  n=30,579 | | | | |  | Women  n=35,296 | | | | | |
| --- | --- | --- | --- | --- | --- | --- | --- | --- | --- | --- | --- | --- |
|  | Smoking status | | | | |  | Smoking status | | | | | |
|  | Current |  | Former |  | Never |  | Current |  | Former |  | Never |  |
| Participants, n (%) | 10,770  (35.2) |  | 7,610  (24.9) |  | 12,199  (39.9) |  | 13,350  (37.8) |  | 8,944  (25.3) |  | 13,002  (36.8) |  |
| *Socioeconomic position* |  |  |  |  |  |  |  |  |  |  |  |  |
| Basic level of education, % | 25.8 |  | 17.9 |  | 11.2 |  | 29.4 |  | 18.8 |  | 13.6 |  |
| Teriary level of education, % | 19.5 |  | 28.6 |  | 42.6 |  | 17.6 |  | 30.7 |  | 42.8 |  |
| Disability pension, % | 4.5 |  | 2.3 |  | 2.4 |  | 7.0 |  | 4.3 |  | 4.3 |  |
| *Health behaviors and indicators* |  |  |  |  |  |  |  |  |  |  |  |  |
| Physically inactive in leisure time^2^, % | 25.1 |  | 20.1 |  | 19.4 |  | 23.7 |  | 16.6 |  | 18.4 |  |
| Alcohol >10 times/ month, % | 10.3 |  | 11.4 |  | 8.4 |  | 4.7 |  | 5.1 |  | 3.3 |  |
| Fruit/vegetables once or more /day, % | 37.1 |  | 50.1 |  | 53.7 |  | 62.7 |  | 77.6 |  | 78.8 |  |
| Fish once or more /week, % | 69.4 |  | 73.8 |  | 77.1 |  | 74.6 |  | 79.5 |  | 82.0 |  |
| Excellent subjective health, % | 14.8 |  | 23.3 |  | 28.4 |  | 20.0 |  | 29.2 |  | 31.1 |  |
| Self-reported CVD^1^, % | 2.5 |  | 2.4 |  | 1.5 |  | 1.5 |  | 1.5 |  | 1.2 |  |
| *Cardiometabolic risk factors* |  |  |  |  |  |  |  |  |  |  |  |  |
| Pulse, beat/minute, mean | 74.3 |  | 69.9 |  | 68.9 |  | 77.3 |  | 73.3 |  | 74.2 |  |
| Serum total cholesterol, mmol/l, mean | 5.8 |  | 5.8 |  | 5.6 |  | 5.5 |  | 5.3 |  | 5.3 |  |
| *Anthropometric measures* |  |  |  |  |  |  |  |  |  |  |  |  |
| Height, cm, mean | 179.0 |  | 179.7 |  | 180.0 |  | 166.1 |  | 166.6 |  | 166.2 |  |
| Weight, kg, mean | 83.6 |  | 87.5 |  | 86.1 |  | 68.2 |  | 70.8 |  | 69.7 |  |
| BMI, kg/m^2^, mean | 26.1 |  | 27.1 |  | 26.6 |  | 24.7 |  | 25.5 |  | 25.2 |  |
| Hip circumference, cm, mean | 101.7 |  | 103.8 |  | 103.2 |  | 99.9 |  | 101.8 |  | 101.4 |  |
| Waist circumference, cm, mean | 90.8 |  | 93.2 |  | 91.8 |  | 78.6 |  | 79.6 |  | 78.8 |  |
| Waist-to-hip ratio | 0.89 |  | 0.90 |  | 0.89 |  | 0.79 |  | 0.78 |  | 0.78 |  |
| Mid-upper arm circumference, cm, mean | 30.8 |  | 31.4 |  | 31.0 |  | 28.2 |  | 28.8 |  | 28.6 |  |

^1^Self-report of myocardial infarction, angina or stroke; ^2^Less than 4 hours leisure-time physical activity per week
