# Supplementary material for: Associations of tobacco smoking with body mass distribution; a population-based study of 65,875 men and women in midlife
Source: BMC Public Health. 2019 Nov 1;19:1439. doi: 10.1186/s12889-019-7807-9 (PMC6825363; doi:10.1186/s12889-019-7807-9)
Supplement: Supplementary file 2 — Additional file 2: Table S2. Mean waist circumference and mean waist-hip ratio by smoking status by levels of leisure-time physical activity. [file 12889_2019_7807_MOESM2_ESM.docx]

Additional file 2: Table S2. Mean waist circumference and mean waist-hip ratio by smoking status

by levels of leisure-time physical activity.

|  | Model 2_SEP and health indicators_ | | Model 3_BMI additionally_ | |
| --- | --- | --- | --- | --- |
| Smoking status | Never | Current | Never | Current |
| *Men, N=22,969* |  |  |  |  |
| *Physically inactive^1^, n* | *2,361* | *2,707* |  |  |
| BMI, kg/m^2^ | 27.4 | 26.3*** |  |  |
| HC, cm | 103.9 | 102.2*** | 103.2 | 103.0^p=0.055^ |
| WC, cm | 94.6 | 92.3*** | 93.4 | 93.5^NS^ |
| WHR | 0.91 | 0.90*** | 0.90 | 0.91* |
| *Intermediate active^2^, n* | *5,879* | *5,279* |  |  |
| BMI, kg/m^2^ | 26.6 | 26.0*** |  |  |
| HC, cm | 103.2 | 101.9*** | 102.7 | 102.3*** |
| WC, cm | 92.1 | 90.8*** | 91.4 | 91.5 ^NS^ |
| WHR | 0.89 | 0.89^NS^ | 0.889 | 0.893*** |
| *Highly active^3^, n* | *3,959* | *2,784* |  |  |
| BMI, kg/m^2^ | 26.2 | 25.7*** |  |  |
| HC, cm | 102.5 | 101.2*** | 102.2 | 101.6*** |
| WC, cm | 89.9 | 89.2** | 89.3 | 89.8*** |
| WHR | 0.876 | 0.880** | 0.87 | 0.88*** |
| *Women, N=26,352* |  |  |  |  |
| *Physically inactive^1^, n* | *2,392* | *3,170* |  |  |
| BMI, kg/m^2^ | 26.4 | 25.2*** |  |  |
| HC, cm | 103.4 | 100.6*** | 102.3 | 101.7*** |
| WC, cm | 82.0 | 79.9*** | 80.7 | 81.2*** |
| WHR | 0.79 | 0.79^NS^ | 0.79 | 0.80*** |
| *Intermediate active^2^, n* | *8,401* | *8,157* |  |  |
| BMI, kg/m^2^ | 25.2 | 24.5*** |  |  |
| HC, cm | 101.4 | 99.6*** | 100.8 | 100.2*** |
| WC, cm | 78.9 | 77.9*** | 78.1 | 78.7*** |
| WHR | 0.778 | 0.783** | 0.78 | 0.79*** |
| *Highly active^3^, n* | *2,209* | *2,023* |  |  |
| BMI, kg/m^2^ | 24.6 | 24.2*** |  |  |
| HC, cm | 100.0 | 98.8*** | 99.7 | 99.2*** |
| WC, cm | 76.9 | 76.9^NS^ | 76.4 | 77.3*** |
| WHR | 0.777 | 0.781*** | 0.77 | 0.78*** |

^1^Sedentary leisure-time activities and hard exercise<1 hour/week; ^2^light physical activities >4 or hard exercise 1-2 hour/week; ^3^intermediate or hard physical activity >3 hours/week.

Model 2 _SEP and health indicators_ adjusted for height, disability pension, and alcohol use; Model 3_BMI additionally_ adjusted in addition for BMI; *p < 0.05, ** p< 0.01; *** p<0.001
