# Supplementary material for: Associations of tobacco smoking with body mass distribution; a population-based study of 65,875 men and women in midlife
Source: BMC Public Health. 2019 Nov 1;19:1439. doi: 10.1186/s12889-019-7807-9 (PMC6825363; doi:10.1186/s12889-019-7807-9)
Supplement: Supplementary file 3 — Additional file 3: Table S3. Mean values of weight- related indices by smoking status by educational attainment. [file 12889_2019_7807_MOESM3_ESM.docx]

Additional file 3: Table S3. Mean values of weight- related indices by smoking status by educational attainment.

|  | Model 2 _SEP and health indicators_ | | Model 3 _BMI additionally_ | |
| --- | --- | --- | --- | --- |
| Smoking status | Never | Current | Never | Current |
| *Men, N=22,969* |  |  |  |  |
| *Basic education, n* | *1,405* | *2,809* |  |  |
| BMI, kg/m^2^ | 27.5 | 26.2*** |  |  |
| HC, cm | 103.6 | 101.5*** | 102.8 | 102.3*** |
| WC, cm | 93.6 | 91.2*** | 92.3 | 92.6^NS^ |
| WHR | 0.90 | 0.90* | 0.897 | 0.903*** |
| *Secondary education, n* | *5,610* | *5,867* |  |  |
| BMI, kg/m^2^ | 26.8 | 26.0*** |  |  |
| HC, cm | 103.3 | 101.8*** | 102.7 | 102.4*** |
| WC, cm | 92.5 | 90.7*** | 91.5 | 91.7 ^NS^ |
| WHR | 0.89 | 0.89** | 0.890 | 0.894*** |
| *Tertiary education, n* | *5,184* | *2,094* |  |  |
| BMI, kg/m^2^ | 26.1 | 25.9^p=0.051^ |  |  |
| HC, cm | 102.8 | 102.1*** | 102.7 | 102.2*** |
| WC, cm | 90.7 | 90.6^NS^ | 90.5 | 90.8* |
| WHR | 0.88 | 0.89* | 0.88 | 0.89*** |
| *Women, N=26,352* |  |  |  |  |
| *Basic education, n* | *1,815* | *3,960* |  |  |
| BMI, kg/m^2^ | 26.6 | 25.1*** |  |  |
| HC, cm | 103.1 | 100.0*** | 101.8 | 101.3*** |
| WC, cm | 82.3 | 79.5*** | 80.6 | 81.2*** |
| WHR | 0.80 | 0.79^NS^ | 0.79 | 0.80*** |
| *Secondary education, n* | *5,651* | *7,049* |  |  |
| BMI, kg/m^2^ | 25.5 | 24.5*** |  |  |
| HC, cm | 101.7 | 99.8*** | 101.0 | 100.6*** |
| WC, cm | 79.4 | 78.2*** | 78.4 | 79.2*** |
| WHR | 0.778 | 0.783** | 0.78 | 0.79*** |
| *Tertiary education, n* | *5,536* | *2,341* |  |  |
| BMI, kg/m^2^ | 24.5 | 24.4^NS^ |  |  |
| HC, cm | 100.5 | 99.6*** | 100.5 | 99.7*** |
| WC, cm | 77.1 | 77.7* | 77.1 | 77.7*** |
| WHR | 0.77 | 0.78*** | 0.77 | 0.78*** |

Model 2 _SEP and health indicators_: adjusted for body height, disability pension, physical activity and alcohol use. Model 3_BMI additionally,_ adjusted for disability pension, physical activity, alcohol use and BMI; *p < 0.05, ** p< 0.01; *** p<0.001
