# Supplementary figures and images for: Associations of tobacco smoking with body mass distribution; a population-based study of 65,875 men and women in midlife
Source: BMC Public Health. 2019 Nov 1;19:1439. doi: 10.1186/s12889-019-7807-9 (PMC6825363; doi:10.1186/s12889-019-7807-9)

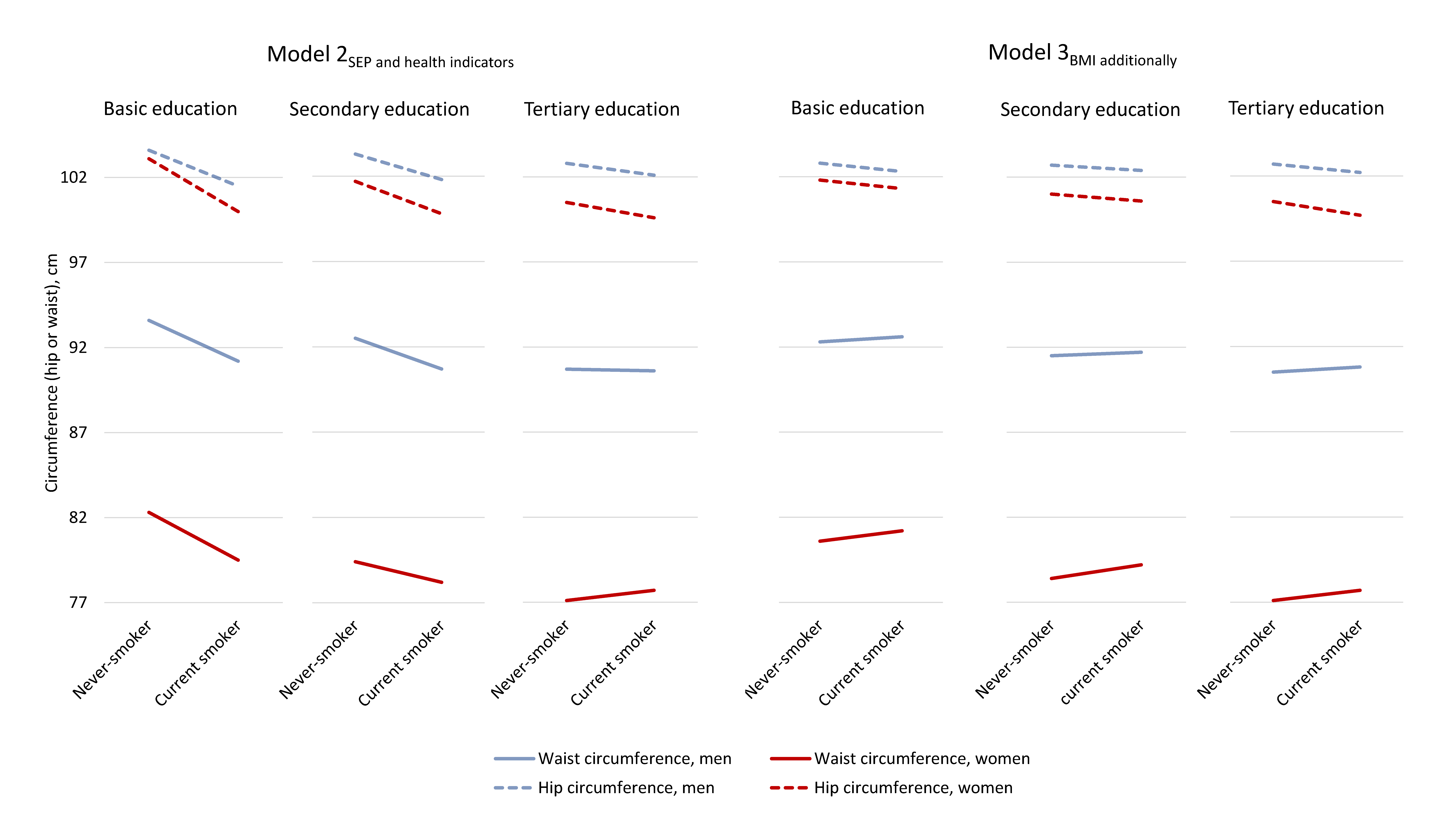

Supplement: Supplementary file 4 — Additional file 4: Figure S1. Adjusted mean hip and waist circumferences by smoking status in strata by education. Based on Additional file 3: Table S3. [file 12889_2019_7807_MOESM4_ESM.tiff]
